# How to write a scientific paper in fifteen steps

**DOI:** 10.1371/journal.pcbi.1013505

**Published:** 2025-09-24

**Authors:** John M. Drake, Barbara A. Han

**Affiliations:** 1 Odum School of Ecology, University of Georgia, Athens, Georgia, United States of America; 2 Cary Institute of Ecosystem Studies, Millbrook, New York, United States of America; SIB Swiss Institute of Bioinformatics, SWITZERLAND

Many scientists, at every career stage, struggle to begin writing scientific papers. This is often not a matter of procrastination or a lack of ideas, but of knowing how to start: what kind of sentence belongs at the beginning of a paragraph, what kind of paragraph belongs at the beginning of a section, and what kind of story emerges from a collection of results. These are fundamentals of writing, but they are often difficult to implement in scientific writing, where the ideas can be complex and the structure less intuitive. In our experience mentoring graduate students and postdocs, we’ve seen that drafts often lack topic sentences, transitions, or a clear rationale—not because the writer lacks good ideas, but because those ideas are not yet scaffolded into a structure that connects them.

**Fig 1:**
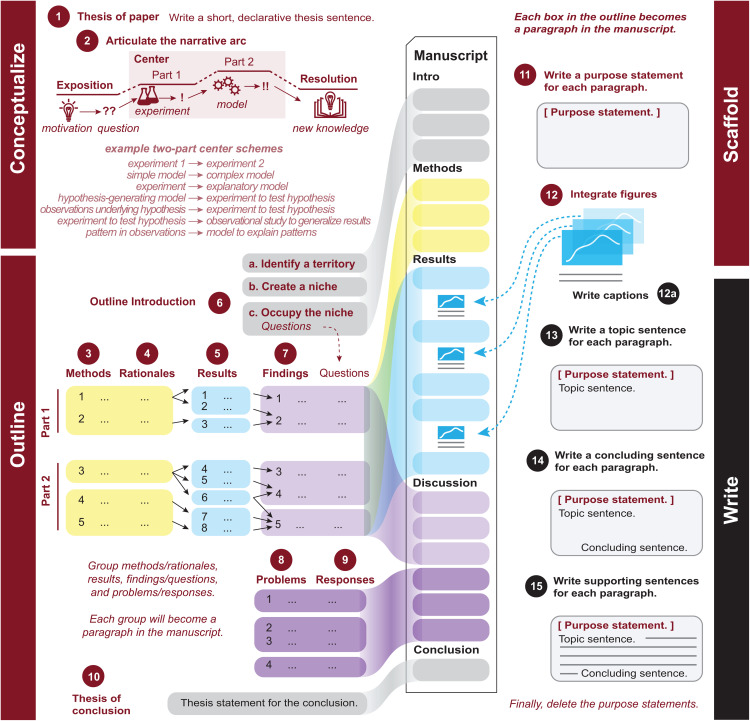
A structured workflow for drafting a scientific paper in 15 Steps. Use this figure as a practical map: work through it sequentially to build a manuscript from scratch, or revisit individual steps to diagnose problems and guide revision.

Our approach differs from the traditional sequence in which papers are read (Introduction, Methods, Results, Discussion) and from the “writing backwards” approach that begins with Results [1]. Instead, our approach follows a logic of discovery rather than a logic of presentation. It begins by defining the core reasoning of the paper before turning to methods, results, and interpretation and thereby separates the work of thinking from the work of phrasing. The “15 Steps” presented here (Fig 1) grew out of attempts to demystify the writing process by offering a clear sequence of conceptually grounded steps that generate the content and structure of a scientific manuscript. While no recipe will work for everyone, this one has proven helpful to many students and early-career scientists. It is designed to support the process of writing and can also be used retrospectively as a tool to diagnose and revise drafts that are structurally underdeveloped.

In the current era, when writing about writing, one should probably address the use of generative AI. Some authors are now turning to tools like ChatGPT to help them overcome the “blank-page problem”. We see promise in these technologies, particularly in the revision and editing stages—clarifying language, suggesting alternative phrasings, and identifying unsupported claims. However, we urge writers to complete the intellectual work of shaping their ideas before relying on generative models to help express them. Effective use of AI requires metacognitive and metalinguistic skills: you must know what you are trying to say, how it might be misunderstood, and how to instruct the tool to help. For novice writers, the risk is not simply factual error or hallucination, but substituting fluency for clear thinking. We therefore suggest that writers follow the 15 Steps before engaging with AI tools, recognizing that these tools may be most helpful at the editing stage—once the hard work of reasoning, framing, and structuring the paper is done.

**Step 1. What is the main point of your study?** Write it as a short declarative sentence. This is the working title. This is the thesis of the paper. Every paragraph must illuminate this thesis. Don’t start writing until you know the main point. Until you know the main point, you have nothing to say.

For instance, the title of one recent paper in *PLOS Computational Biology* is “Dose-dependent interaction of parasites with tiers of host defense predicts wormholes that prolong infection at intermediate inoculum sizes” [2]. This is a great thesis. It has both a subject (“interaction of parasites with tiers of host defense”) and a verb (“predicts”). It scopes the phenomenon of interest (duration of infection). It supplies the focus needed to dismiss tangents, keeping every part of the paper oriented toward a single, unifying insight. Note that a working thesis need not double as the final title. While the thesis anchors the paper’s reasoning, a published title serves additional functions such as attracting interest while signaling scope. It might even be a question.

**Step 2. Determine what the *story* is.** Articulate the *narrative arc*. Answer such questions as: What did we want to learn? Why did we need to learn this? What did we do? What do we know now? Sketch it out as a flow chart. The purpose of this sketch is to help us keep in view *where we are going*, to avoid thinking in circles or stuck in a logical cul-de-sac. The arc follows key transitions that structure how knowledge is built—for example, from question to answer, from theory to evidence, or from data to interpretation. A common arc might begin with a prediction generated by a simple model, followed by an experiment to empirically test the prediction. The centerpiece of the narrative should consist of two parts. If it contains only one part, it may be too simple to be interesting (or publishable). If it contains three or more parts, it may be too complicated to remember (or it may need to be a monograph). Ergo, in most cases, a two-part narrative arc will be most effective. Parts are typically one of the following: experiment, model, observational study, meta-analysis. Example *two-part schemes* include the following. With relatively little imagination, this list can be extended considerably.

**Table:** 

Part 1	Part 2
Experiment 1	Experiment 2
Simple model	Complicated model
Experiment	Model to explain experimental observations
Model to generate hypothesis	Experiment to test hypothesis
Observations underlying a hypothesis	Experiment to test hypothesis
Experiment to test hypothesis	Observational study generalizing experimental results
Pattern in observations	Model to explain pattern

Two examples illustrate Step 2. The two-part arc in Kirk and colleagues [3] pairs experimental data collection with a theory-driven modeling approach. Their motivating question was whether the Metabolic Theory of Ecology (MTE)—which posits that metabolic rate, as a function of body size and temperature, governs ecological patterns from individuals to ecosystems—could serve as a predictive framework for within-host parasite dynamics. This matters because temperature affects both host defenses and parasite replication, and forecasting infection outcomes under climate change requires a general, mechanistically grounded model. In the first part of the paper, they measured host survival and parasite burden across a temperature gradient in a model system (*Daphnia*–*Ordospora*). In the second part, they modeled these dynamics using temperature-dependent rate equations derived from MTE, and showed that the model accurately captured the costs of infection across the thermal range.

In Nuismer and colleagues [4], a two-part structure combines general theory with an applied case study. Their central question was whether programs that reduce zoonotic spillover, which are typically regarded as public health victories, might paradoxically increase disease burden in the long term. In the first part, they developed an age-structured model of infection in which disease severity increases with age at first exposure. In the second, they parameterized their model using empirical data on Lassa virus in West Africa and identified conditions under which spillover reduction could backfire (when immunity is long-lasting and spillover pressure is very high).

A clearly articulated question, tied to a consequential problem, helps make the arc intelligible and relevant. Choosing the right structure is both a storytelling device and helps to shape the logic of the paper and define the scope of its contribution.

**Step 3. What did you do?** Write a numbered list of methods, *e.g.*, “rarefaction was performed on all samples for which species had been identified”. You will be tempted to begin writing in paragraphs. Resist the temptation! There will be time for that later (in Step 15, to be specific). When scripting environments like R or Python are used for analysis, portions of this list often mirror the code itself, offering both a reproducible scientific workflow and a ready-made template for this section [5].

**Step 4. Why did you do what you did?** For each item in (3), declare the rationale with an *infinitive clause* (a “to-clause”) as in, “To estimate species richness...” Combine with the lists from (3) (“To estimate species richness, rarefaction was performed on all samples for which species had been identified”). Each linked pair is a method. Group these methods. These groups will grow up to be paragraphs (in the methods).

**Step 5. What were the results of what you did?** Results are the atoms of which new knowledge is built. Results come from doing things, so every result must have a counterpart in Step 3, and every method must lead to a result. Write a numbered list of results. Every resulting observation, measurement, statistic, etc., should be on a separate line. Group these results. These groups will also grow up to be paragraphs (in the results). Often, there is some iteration between Steps 3, 4, and 5.

For example, here are some results from [3]:

- None of the exposed individuals at 6.0, 9.5, or 33.3 °C became infected, while infection prevalence among exposed individuals at intermediate temperatures ranged from 28% to 97%
- Lifespan was greatest at 11.8 °C for both unexposed and exposed individuals
- Equilibrium parasite abundance decreased sharply at temperatures above 20 °C

These results stem directly from specific methods (e.g., temperature-controlled exposures, lifespan tracking, parasite quantification) and exemplify the kind of discrete, empirical observations that should be itemized at this stage. They are clear, unembellished, and ready to be grouped into paragraphs later.

**Step 6. Outline introduction according to Swales’s CARS model.** CARS stands for “Create A Research Space”. The CARS model consists of three moves [6,7]:

1. Establish a “territory”;
2. Create a “niche”;
3. Occupy the niche.

Mastering this model is crucial because an effective research space determines whether readers will recognize the value of your work. The territory is the general intellectual context in which your work is situated, such as the effects of global change on biogeography or the role of skin microbiome in immune defenses. Establishing the territory typically occurs through three steps: (i) claiming centrality, (ii) making topic generalizations, which are broad, generally accepted facts about the subject; and (iii) reviewing prior work. The niche locates your work within this space. A well-articulated niche captures attention by demonstrating not just what you studied, but why it matters to the broader scientific community. When we fail to establish this space clearly, readers may miss the forest for the trees—seeing our methods and results without grasping their implications or contributions. Conversely, when we excel at creating this space, we transform specialized findings into work that resonates across disciplinary boundaries, increasing both readership and impact. Feel free to throw some elbows (metaphorically speaking) to make a gap that you can step into. Einstein said one thing. Darwin said something else. But no one has looked at *this*. Finally, step into the space you have created. This should culminate in a clear statement of your study’s objectives or hypotheses, which serve to link the introduction with the methods and results that follow. There are several ways to do this, but here are two tried and true approaches. First, simply report on *what you did*. We collected these data. We developed this model. We performed some analysis. The second approach is to write an intellectual summary that ever so briefly foreshadows your findings and places them squarely in the space you just created. This approach usually starts with the phrase “Here we show…”

Write down the territory, niche, and occupying strategy. For ways to do this and some formulaic phrases that might be useful, see *Academic Writing for Graduate Students* [6] and chapter 7 of *Genre Analysis* [7].

**Step 7. Draft a list of findings.** Findings are the interpretation of results. Findings must pertain to the problems outlined in the introduction. Findings are the results of Step 5 placed in context. The relation of results to findings does not need to be one-to-one. In fact, many times a finding will build on several results considered together. However, there cannot be findings unsupported by results or results that do not lead to findings. One device for checking this is to place the lists of results and findings next to each other and then draw a line for each connection between result and finding. Any result or finding left unconnected must be examined to determine if it (i) is irrelevant (in which case delete it), (ii) requires a new item to be added to the opposite list (in which case add it), or (iii) may be connected to an existing item in the opposite list. Group findings. This is the outline of the *first half of the discussion*.

For example, here are two findings from [4], which synthesize multiple results into higher-level interpretations relevant to their central question:

- “Negative public health impacts of spillover reduction are generally unlikely”
- “Negative impacts are possible if disease severity increases sufficiently rapidly with age”

They do not qualify as results, since they are already embedded in a broader interpretive frame—in this case, a presumed public health application; contextualization might instead involve theoretical framing, cross-study comparison, or practical relevance. Additionally, each draws on multiple results (model solutions, sensitivity analysis, etc.) to articulate what the results mean and why they matter.

**Step 8. Draft a list of *problem items***. Findings must be contextualized. Problem items are caveats, inconsistencies, and anticipated objections to findings (the interpretation of results), the results themselves, or the rationale that links the methods with the niche identified in Step 6. This is an excellent occasion to articulate statistical assumptions and identify model limitations. Write the problem items as a numbered list.

Continuing with [4]: “None of the populations for which we could find seroprevalence estimates are predicted to experience a force of spillover sufficient for spillover reductions to lead to negative health consequences,” but “populations may well exist where the contemporary force of Lassa virus spillover exceeds the threshold value required for negative consequences.” This is to say that the authors’ findings apply for all populations for which they could obtain data, but it is plausible that there are populations to which their findings would not apply. This data gap limits the generality of the findings. Later in the same paper: “Although our model is quite general, it makes a range of assumptions.” These assumptions are then itemized (e.g., absence of human-to-human infection, force of spillover is equal across age classes, etc.).

**Step 9. For each problem item provide a *response*.** Typically, a response is a qualification, answer, or synthesis. Problem items and responses are one-to-one. List the problem items and responses together as ordered pairs. Group these pairs. These outline the *second half of the discussion*.

From [4] again, we have these pairs (among others):

**Table:** 

Model assumes absence of human-to-human infection	Results are likely to apply to cases where human-to-human transmission exists but remains minimal
Model assumes force of spillover is equal across age classes	Alert readers to conditions under which assumption is violated to avoid improper generalization
Model assumes human populations have reached steady state	Alert readers to empirical evidence contradicting this assumption

To us, the first of these responses is fully satisfactory, and the second is quite helpful in that it restricts the findings to a clear set of suitable cases, but the third falls short, as it notes a potential issue without assessing how strongly it might affect the conclusions.

**Step 10. Determine the thesis of the closing paragraph.** The closing paragraph is important enough that we treat it by itself. Draft a thesis for this paragraph. The thesis may be a new direction from the thesis of the paper, but the connection should be clear. To reiterate: the connection between the thesis of the paper and the thesis of the final paragraph must be spelled out in the final paragraph.

We like this topic sentence of the closing paragraph of [4]: “Reducing the spillover of zoonotic pathogens holds incredible promise for improving human health.” It is clear and direct; substantive; forward-looking; tied directly to the findings of the paper, but generalizing; and asserts the significance of the paper.

There are a few additional rules for final paragraphs:

- End on a positive note.
- Avoid ending with a caveat or “need for further research”.
- Good endings might be: (i) declaration of main finding or restatement of thesis; (ii) statement of a new question, or (iii) identification of a concrete application.

**Step 11. Find the paragraphs**. The first three paragraphs of your article come from the three moves of the Introduction (Step 6). The rest follow from the groups of methods, groups of results, findings, problem item-response pairs, and the final paragraph. Write a *purpose statement* (one sentence) articulating the role of each paragraph in the narrative arc advancing the thesis of the article. For instance: “This paragraph explains why we used Cormack–Jolly–Seber models rather than band recovery models to estimate population abundance.” These sentences are key. They provide bones and muscle to the narrative arc—bones to hold it in place and give it shape, muscle to move it along. Place these sentences in boldface type before each paragraph. They are not part of the manuscript, but aren’t to be deleted until the manuscript is in its final form. These sentences provide reminders of the logical flow and purpose of each paragraph.

**Step 12. Blend figures with the narrative.** Figures are visual representations of your key data, concepts, or findings that enhance understanding and provide evidence for your thesis when effectively integrated with your text. We make a strong distinction between planning research, conducting research, and reporting on research, and we create figures when we are planning research (*e.g.*, conceptual figures) and conducting research (*e.g.*, plots of data, statistics, or simulation results), so the figures exist before writing commences. These existing figures can always be refined during revision, but having them in place early provides structural scaffolding for the narrative.

Most figures belong in the Results section, although occasionally a conceptual figure may strengthen the Introduction or a study diagram may clarify the Methods. To proceed, first order figures according to the narrative arc. Does each figure illuminate the thesis? If not, strike it. Next, write a caption for each figure. Captions have a few rules:

- The topic sentence of each caption should also be readable as a figure title. And, it should indeed be a sentence—with a subject and a verb—not a label.
- The sequence of captions should be an abbreviation of the narrative, broadly readable without reference to the main text.
- The sequence of topic sentences of captions should be an abbreviation of the narrative, broadly readable without reference to the main text.
- Each caption should contain whatever technical information is needed to understand every aspect of the figure (error bars, units, parameter values, statistical tests, etc.).

Guidance on figure style is beyond the scope of this essay. We suggest consulting [8–10]. *The Visual Display of Quantitative Information* is a timeless classic [11].

**Step 13. Write a topic sentence for each paragraph.** Sometimes these topic sentences may be the same as the purpose statements from Step 11. Other good devices are:

- A question. (“Why is silica limiting to *Cyclotella*?”)
- A turning point. (“Having established the importance of interannual variability in this system, we turn to the problem of coexistence.”)
- A complication. (“This argument suggests that species A should be more abundant than species B in dry years but not in wet years, but it fails to account for temporary migration of mobile adults.”)
- A development. (“This idea can be made more concrete with a model.”)

Does each paragraph support the paper’s main point? If not, strike it or rewrite it.

**Step 14. Write a concluding sentence for each paragraph.** This sentence should close the theme or problem announced in the topic sentence. If the topic sentence is a declaration, the concluding sentence should be a development, clarification, or summary of the support for the declaration. If the topic sentence is a question, this sentence should be a statement of the answer or a statement of why the answer cannot be provided. If the topic sentence and concluding sentence are not connected or do not support the purpose of the paragraph, one or both should be rewritten. The concluding sentence should initiate the segue to the next paragraph, at least in logic if not in syntax. (If the two paragraphs are disconnected, they should be placed in separate subsections).

**Step 15. Write the supporting sentences.** Typically, there should be 3–6 supporting sentences per paragraph. Equations, algorithms, and code snippets serve the same role as supporting sentences and should be written now. Supporting sentences in the paragraph that do not advance the paragraph’s purpose should be deleted. This is key. Do not write a sentence that does not advance the paragraph’s purpose. If you do, strike it. For the methods, results, and conclusions, many of these sentences can be lifted directly from the numbered lists in Steps 4, 5, and 9. Swales [7] provides many examples of supporting sentences in introduction paragraphs. This is also the moment to situate the work within the broader scientific conversation—i.e., to connect the findings to existing studies, highlight consistencies or differences with prior work, and in so doing, show how the contribution advances or reframes our thinking.

The 15 Steps outlined here provide a structured framework for writing scientific papers. They are meant to guide writers from initial ideas through a complete draft, with particular attention to clarity, coherence, and logical flow. Writers can follow them sequentially, or use them as a checklist to revise and strengthen existing drafts. The steps are flexible enough to accommodate different writing styles and scientific domains, but specific enough to support progress when writing feels difficult. We encourage readers to try this approach—not only as a way to get started, but as a way to improve their reasoning, sharpen their message, and produce writing that others can understand and build upon.

Students may wonder when to start writing. We advise beginning only after the research is done. While writing lab notes, written protocols, and documented workflows is essential to clarify thinking during research, the manuscript itself is best begun with a clean slate. Starting too early risks reinforcing preliminary interpretations, creating cognitive inertia, and diverting attention from discovery toward justification. It can also lead to inefficiencies: circling through draft after draft as results evolve, cutting off potentially useful false starts, and incurring high switching costs between analysis and writing. It is better to treat the manuscript as a retrospective.

These 15 Steps differ from some other well-known approaches like writing backwards [1], Lippi’s flowchart [12], and traditional guides to scientific writing [13,14]. We like the 15 Steps because it is detailed and procedural, because thinking about methods before results emphasizes the acts of research rather than rational reconstruction [15], and because it ensures tight and tidy connections between what research was done (methods) and what it means (findings). Similarly, while some might view Steps 1–12 as outlining rather than writing, we don’t find the distinction to be helpful but rather regard the act of formulating ideas in language, even in list form, as central to the writing process.

Writing is a skill, and like any skill, it improves with practice. Our hope is that these steps make that practice a little more systematic—and a little less daunting. We believe the heart of good scientific writing lies in these 15 Steps—everything else is editing.
